# Effect of metformin monotherapy on cardiovascular diseases and mortality: a retrospective cohort study on Chinese type 2 diabetes mellitus patients

**DOI:** 10.1186/s12933-015-0304-2

**Published:** 2015-10-09

**Authors:** Colman Siu Cheung Fung, Eric Yuk Fai Wan, Carlos King Ho Wong, Fangfang Jiao, Anca Ka Chun Chan

**Affiliations:** Department of Family Medicine and Primary Care, Li Ka Shing Faculty of Medicine, The University of Hong Kong, 3/F Ap Lei Chau Clinic, 161 Main Street, Ap Lei Chau, Hong Kong

**Keywords:** Diabetes mellitus, Metformin, Cardiovascular disease, Mortality, Diabetes care

## Abstract

**Background:**

Many factors influence whether the first-line oral anti-diabetic drug, metformin, should be initiated to a patient with type 2 diabetes mellitus (T2DM) early in the course of management in addition to lifestyle modifications. This study aims to evaluate the net effects of metformin monotherapy (MM) on the all-cause mortality and cardiovascular disease (CVD) events.

**Methods:**

A retrospective 5-year follow-up cohort study was conducted on Chinese adult patients with T2DM and without any CVD history under public primary care. Cox proportional hazard regressions were performed to compare the risk of all-cause mortality and CVD events (CHD, stroke, heart failure) between patients receiving lifestyle modifications plus MM (MM groups) and those with lifestyle modifications alone (control groups).

**Results:**

3400 pairs of matched patients were compared. MM group had an incidence rate of 7.5 deaths and 11.3 CVD events per 1000 person-years during a median follow-up period of 62.5 months whereas control group had 11.1 deaths and 16.3 per 1000 person-years during a median follow-up period of 43.5–44.5 months. MM group showed a 29.5 and 30–35 % risk reduction of all-cause mortality and CVD events (except heart failure) than control group (P < 0.001). MM group was more prone to progress to chronic kidney disease but this was not statistically significant.

**Conclusions:**

Type 2 diabetic patients who were started on metformin monotherapy showed improvement in many of the clinical parameters and a reduction in all-cause mortality and CVD events than lifestyle modifications alone. If there is no contraindication and if tolerated, diabetic patients should be prescribed with metformin early in the course of the diabetic management to minimize their risk of having the cardiovascular events and mortality in the long run.

**Electronic supplementary material:**

The online version of this article (doi:10.1186/s12933-015-0304-2) contains supplementary material, which is available to authorized users.

## Background

Diabetes mellitus (DM) is a major public health issue as it is estimated that 1 in 12 people are affected by diabetes in the world [1]. The World Health Organization has projected that by 2030, diabetes will be the seventh most common cause of death in the world [2]. Good control of DM is crucial because DM is closely linked to various complications, ranging from different cardiovascular diseases (including myocardial infarction, cerebrovascular diseases, etc.) to microvascular diseases like diabetic retinopathy [including sight-threatening diabetic retinopathy (STDR)] and diabetic nephropathy [including end stage renal failure (ESRF)].

Primary care is experiencing more pressure as the numbers of patients with diabetes mellitus increase dramatically. Many type 2 diabetic patients are under the care of primary care doctors especially if they are newly diagnosed with DM or if their condition is relatively stable. Sometimes, primary care doctors or family physicians have a difficult time deciding if they should initiate oral anti-diabetic (OAD) drugs to patients, as there are many factors influencing whether a drug should be initiated, despite some evidence suggesting that early use of OAD drugs, like metformin, is beneficial to the patients in terms of lowering the clinical parameters like haemoglobin A1c (HbA1c), or reduction of DM-related complications or mortality [3]. Metformin, the only available biguanide amongst the OAD drugs, is widely used as the first-line OAD in the management of diabetic patients because of the longstanding evidence of its efficacy and comparatively less side-effects like hypoglycaemia than sulphonylurea group of OAD drugs [4]. Some studies have even advocated the early use of metformin as it was found to delay or prevent the incidence of DM or DM-related complications [5]. However, patients may prefer not to start OAD drugs for various reasons, including possible side-effects, compliance issues, life-long drug therapy etc. These concerns are not limited to the Chinese population, but are worldwide [6–8]. One of the hypotheses was that since DM was a chronic disease closely linked to lifestyle (including dietary habit and exercise intensity), control of these factors, or the lifestyle modifications should be implemented before the use of OAD drugs [9–13]. American Diabetic Association Guidelines suggested metformin, if not contraindicated and if tolerated, is the preferred initial pharmacological agent for type 2 DM [14] while the local Hong Kong Reference Framework for Diabetes Care for Adults in Primary Care Setting 2010 suggested initiating metformin as a monotherapy for HbA1c <7.5 % for diabetic patients [15]. In obese patients, metformin-based monotherapy reduced cardiovascular events compared to patients treated with lifestyle modifications alone [16]. Despite the availability of these guidelines or reference frameworks, the decision to start metformin is complex issue and involves clinical judgement. There is still a significant proportion of diabetic patients who have not started on any anti-diabetic medication [17–19]. To our knowledge, there are no previous studies on the direct comparison between lifestyle modifications alone verses metformin monotherapy (MM) in addition to lifestyle modifications on the outcomes of Chinese diabetic patients. Thus, this study aims to evaluate the net effectiveness of MM on the diabetes-related cardiovascular complications and mortality in Chinese patients diagnosed with type 2 diabetes by comparing patients with lifestyle modifications alone and patients with lifestyle modifications plus MM.

## Methods

### Study design

This was a retrospective cohort study on patients with type 2 diabetes under primary care and the dataset was extracted from a large scale local diabetic programme [20]. The data were collected from the diabetic patients under primary care outpatient clinics managed by the Hong Kong Hospital Authority (HA) across the whole territory between 1 August 2008 and 31 December 2008. The data were made available from a large scale study for the evaluation of local diabetic programme [20]. The HA is the largest government organization monitoring all publically-funded hospitals and outpatient clinics in Hong Kong.

Chinese patients aged 18 years or older with a clinical diagnosis of type 2 diabetes, no history of Cardiovascular Diseases (CVD), and receiving care with MM or lifestyle modifications in primary care clinics of HA between 1 August 2008 and 31 December 2008 were included in the study. According to British National Formulary, metformin was not recommended for use in diabetic patients with estimated glomerular filtration rate (eGFR) <30 ml/min/1.73 m^2^ [21] and thus patients with eGFR <30 ml/min/1.73 m^2^ were excluded in the study. The clinical diagnosis of type 2 diabetes was defined with the International Classification of Primary Care-2 (ICPC-2) code of ‘T90’ through the administrative database of HA and the CVD identification was showed in the section below. Patients in MM group were defined as patients who were prescribed metformin as their sole anti-DM drugs at baseline (regardless if it was newly prescribed or continuation of prescription), and they were excluded if an additional anti-DM drug was added or if they switched to another class of OAD drug or insulin, or stopped metformin within 1 year after the baseline, while patients in control group were defined as patients not using any of the anti-DM drugs at baseline (regardless if they had never been prescribed anti-DM drugs or if they were previously prescribed anti-DM drugs but were stopped for whatever reasons), and they were excluded if any anti-DM drug was added within 1 year after the baseline. Patients who developed any of the outcome events within 1 year after the baseline were also excluded for both arms. The baseline dates for MM and control groups were defined as the date of first prescription with metformin only and the first attendance record in primary care clinics for DM follow-up, respectively. There were no standardized definition or protocol of lifestyle modifications in the care plan for diabetic patients in primary care, however, all diabetic patients in both groups received personal lifestyle advice and counselling from the attending doctors and the nurses during their usual DM follow-up consultation care.

### Exposures

Follow-up began at baseline and continued until either the anti-diabetic drug was switched or an additional anti-diabetic drug was added, the date of incidence of outcome, all-cause mortality, a censoring event or last contact with any inpatient and outpatient services of HA or 31 December 2013. For diabetic patients, who are under the care of primary care physicians/family doctors in the primary care clinics of HA, there is usually a 3 to 4 months (12–16 weeks) time period between each follow-up consultation, and the patient will be prescribed enough chronic medications (including anti-diabetic drugs) to last the period between the follow up consultations. The doctors seldom write a prescription that provides medication for a period of over 120 days. For diabetic patients, who do not need any chronic medications, the period between the follow up consultations may be longer but will not be more than 6 months (180 days). Thus, the censoring events were no drug prescription record for 121 days and no attendance record in primary care clinics of HA for 181 days, for MM group and control group, respectively.

### Outcomes: cardiovascular and renal events and mortality

The outcomes of interest were the following six events: (1) CVD event with any one of the following diagnoses: (i) coronary heart disease (CHD), (ii) stroke, or (iii) heart failure, (2) CHD, (3) stroke, (4) heart failure, (5) all-cause mortality and (6) severe chronic kidney disease (CKD). The comorbidities were identified by the diagnosis coding system of ICPC-2 and International Classification of Diseases, Ninth Edition, Clinical Modification (ICD-9-CM). Hong Kong has a highly subsidized public health care therefore the HA serves the majority of patients with chronic disease, such as those with DM, providing 90 % of in-patient service and covering around 86 % of the total hospital admissions in the whole territory [22, 23]. Most of these patients’ event incidence were captured by the HA central database.

The time of CHD including ischaemic heart disease, myocardial infarction, coronary death and sudden death was taken as the earliest date of diagnosis with ICPC-2 of K74 to K76 or ICD-9-CM of 410.x, 411.x to 414.x, 798.x. The time of heart failure was taken as the earliest date of diagnosis with ICPC-2 of K77 or ICD-9-CM of 428.x. The time of stroke including fatal and non-fatal was taken as the earliest date of diagnosis with ICPC-2 of K89 to K91 or ICD-9-CM of 430.x to 438.x. All-cause mortality were determined using the Hong Kong Death Registry population data. Severe CKD was defined as eGFR <30 ml/min/1.73 m^2^.

### Baseline covariates

The baseline covariates included patient’s socio-demographics, clinical parameters, disease characteristics and treatment modalities. Socio-demographics of patients included age, gender, smoking status, drinking habit and education level. Clinical parameters comprised body mass index (BMI), HbA1c, systolic and diastolic blood pressure (SBP & DBP), lipid profile [low-density lipoprotein-cholesterol (LDL-C) and total cholesterol to high-density lipoprotein-cholesterol (TC/HDL-C) ratio and triglyceride (TG)]. Disease characteristics included self-reported duration of diabetes mellitus (<5 years; 5–10 years and >10 years) and hypertension which was defined as the clinical diagnosis with ICPC-2 code of “K86” or “K87”. The stage of CKD at baseline was classified according to the eGFR (Stage 1: eGFR ≥90 ml/min/1.73 m^2^; Stage 2: eGFR ≥60 ml/min/1.73 m^2^ and <90 ml/min/1.73 m^2^; Stage 3: eGFR ≥30 ml/min/1.73 m^2^ and <60 ml/min/1.73 m^2^). Treatment modalities included the baseline use of anti-hypertensive drug and lipid-lowering agent. All laboratory assays were performed in accredited laboratories by the College of American Pathologists, the Hong Kong Accreditation Service or the National Association of Testing Authorities, Australia. The record of all risk factors closest to baseline for each patient was used.

### Data analysis

Descriptive statistics were used to summarize the baseline characteristics of demographic and clinical parameters, disease characteristics and treatment modalities. Differences in baseline characteristics between MM and control groups were tested using independent t tests for continuous variables or Chi-square tests for categorical variables. The change in clinical parameters including HbA1c, SBP, DBP, LDL-C, TC/HDL-C ratio, TG and BMI after baseline between MM group and control group were examined using independent t tests and the change in stage of CKD after baseline between MM group and control group was evaluated using Chi-square tests.

The incidence rate of CVD was estimated by an exact 95 % confidence interval (CI) based on a Poisson distribution [24]. Kaplan–Meier survival curves were reported and the survival rate differences between groups were compared using the log-rank test. Multivariable Cox proportional hazards regression accounting for all baseline covariates was used to evaluate the effect of MM group comparing with control group on the outcomes. Hazard ratio (HR) and its 95 % confidence intervals were reported for each variable in the regression models. The proportional hazards assumption was assessed by examining plots of the scaled Schoenfeld residuals against time for the predictors. Presence of multi-collinearity was also checked by examining the variance inflation factor. The accuracy of the models was evaluated using Harrell’s discrimination C-index, ranging from zero to one. A value of 0.5 indicates no predictive discrimination, and values of 0 or 1.0 indicate perfect separation of patients [25].

The analysis was repeated on the cohort using propensity score matching analysis. The aim of propensity score matching analysis was to select patients from MM and control groups with similar baseline characteristics. The propensity score modelled the probability of metformin using multivariable logistic regression adjusted by all baseline covariates. The propensity score mapping was made using a one-to-one matching with the nearest neighbour, within 0.001 caliper and without replacement approach. Moreover, two separate sensitivity analyses were performed by (1) considering patients with the exclusion of outcome events occurring within 1 year after study began; and (2) using intention-to-treat approach with patients staying in the original group regardless of treatment continuation, switching or titration and using “last contact” instead of “censoring events” as the end of follow-up.

All significance tests were two-tailed and those with a p value less than 0.05 were considered statistically significant. All statistical analyses were performed using STATA Version 13.0.

Collection and analyses of the data in this study was ethically approved by all local Institutional Review Board (the University of Hong Kong/Hospital Authority Hong Kong West (UW 10-369), Hong Kong East (HKEC-2010-093), Kowloon East and Kowloon Central (KC/KE-10-0210/ER-3), Kowloon West (KW/EX/10-317 (34-04)), New Territories East (CRE-2010.543), and New Territories West clusters (NTWC/CREC/1091/12)) and clinical trial registry (NCT02034695, ClinicalTrials.gov).

## Results

**Fig. 1:**
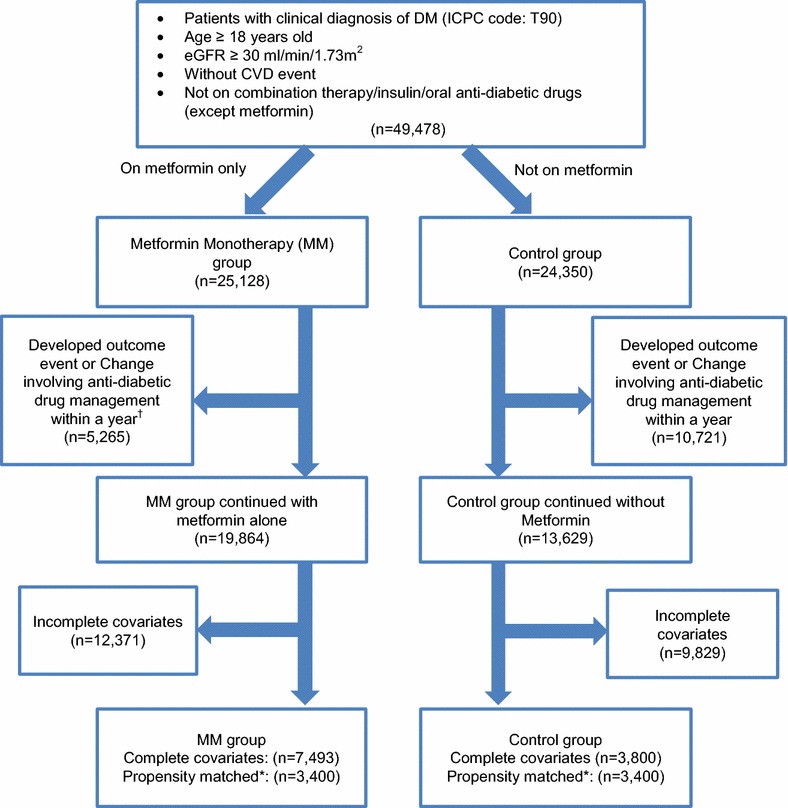
Flow chart of subjects matching and comparison. *MM* metformin monotherapy, *CVD* cardiovascular disease

The flow of diabetic subjects in the study was summarized in Fig. 1. Originally, there were 25,128 (50.8 %) and 24,350 (49.2 %) eligible Chinese patients in MM and control group under the primary care of HA across the whole territory between 1 August 2008 and 31 December 2008, respectively. With the exclusion of those subjects whose management plan changed within 1 year from baseline (e.g. adding or switch to another class of anti-diabetic drug) and incomplete data of baseline covariates, subjects remaining in MM and control groups were 7493 and 3800 respectively. Finally, 3400 subjects from each group were matched with each other using propensity score matching regarding to all baseline characteristics.

Table 1 displays the baseline characteristics between MM and control groups before and after propensity score matching. In general, MM group was younger and had a larger proportion of smokers than control group. Also, most of the clinical characteristics between the two groups were significantly different. MM group had significantly higher HbA1c, triglyceride, BMI and longer duration of DM and larger proportion of subjects with usage of lipid-lowering agents. On the other hand, control group had higher SBP, LDL-C, TC/HDL-C ratio, and had a larger proportion of subjects with hypertension, usage of anti-hypertensive drugs, and Stage 2 or above in CKD staging. After propensity score matching, the two matched groups showed no difference in terms of baseline characteristics.

**Table 1:** Baseline characteristics between metformin monotherapy and control groups before and after matching

Factor	Full cohort with complete case				Propensity score-matched cohort			
	Total (N = 11,293)	MM group (N = 7493)	Control group (N = 3800)	P value	Total (N = 6800)	MM group (N = 3400)	Control group (N = 3400)	P value
Socio-demographic (%, n)								
Age (mean ± SD, n), years	62.05 ± 10.83 (11,293)	61.70 ± 10.75 (7493)	62.75 ± 10.97 (3800)	<0.001*	62.57 ± 10.78 (6800)	62.64 ± 10.58 (3400)	62.51 ± 10.98 (3400)	0.621
Gender				0.728				0.711
Female	59.51 % (6721)	59.63 % (4468)	59.29 % (2253)		59.31 % (4033)	59.09 % (2009)	59.53 % (2024)	
Male	40.49 % (4572)	40.37 % (3025)	40.71 % (1547)		40.69 % (2767)	40.91 % (1391)	40.47 % (1376)	
Smoking status				<0.001*				0.878
Non-smoker	92.69 % (10,468)	91.82 % (6880)	94.42 % (3588)		94.04 % (6395)	94.09 % (3199)	94.00 % (3196)	
Smoker	7.31 % (825)	8.18 % (613)	5.58 % (212)		5.96 % (405)	5.91 % (201)	6.00 % (204)	
Alcohol status				0.990				0.634
Non-drinker	84.77 % (9573)	84.77 % (6352)	84.76 % (3221)		85.03 % (5782)	85.24 % (2898)	84.82 % (2884)	
Drinker	15.23 % (1720)	15.23 % (1141)	15.24 % (579)		14.97 % (1018)	14.76 % (502)	15.18 % (516)	
Educational level				0.235				0.672
No formal education/primary	60.23 % (6802)	59.84 % (4484)	61.00 % (2318)		61.04 % (4151)	61.29 % (2084)	60.79 % (2067)	
Secondary/tertiary	39.77 % (4491)	40.16 % (3009)	39.00 % (1482)		38.96 % (2649)	38.71 % (1316)	39.21 % (1333)	
Clinical parameters (Mean ± SD)								
HbA1c, %	6.83 ± 0.96 (11,293)	6.99 ± 1.05 (7493)	6.52 ± 0.64 (3800)	<0.001*	6.57 ± 0.64 (6800)	6.57 ± 0.67 (3400)	6.58 ± 0.62 (3400)	0.351
SBP, mmHg	133.17 ± 16.91 (11,293)	132.92 ± 17.02 (7493)	133.67 ± 16.69 (3800)	0.026*	133.41 ± 16.77 (6800)	133.28 ± 16.92 (3400)	133.54 ± 16.62 (3400)	0.512
DBP, mmHg	74.94 ± 10.04 (11,293)	74.98 ± 10.08 (7493)	74.84 ± 9.98 (3800)	0.479	74.75 ± 9.97 (6800)	74.68 ± 10.00 (3400)	74.83 ± 9.95 (3400)	0.530
LDL-C, mmol/L	3.25 ± 0.84 (11,293)	3.18 ± 0.82 (7493)	3.40 ± 0.87 (3800)	<0.001*	3.34 ± 0.84 (6800)	3.32 ± 0.83 (3400)	3.35 ± 0.85 (3400)	0.106
TC/HDL-C Ratio	4.36 ± 1.26 (11,293)	4.31 ± 1.13 (7493)	4.45 ± 1.46 (3800)	<0.001*	4.39 ± 1.16 (6800)	4.38 ± 1.13 (3400)	4.41 ± 1.18 (3400)	0.327
Triglyceride, mmol/L	1.65 ± 0.89 (11,293)	1.68 ± 0.91 (7493)	1.59 ± 0.85 (3800)	<0.001*	1.60 ± 0.83 (6800)	1.60 ± 0.78 (3400)	1.60 ± 0.87 (3400)	0.914
BMI, kg/m^2^	25.63 ± 3.91 (11,293)	25.69 ± 3.89 (7493)	25.51 ± 3.94 (3800)	0.018*	25.59 ± 3.85 (6800)	25.56 ± 3.72 (3400)	25.61 ± 3.98 (3400)	0.665
Stage of CKD (%, n)				<0.001*				0.711
Stage 1	35.74 % (4036)	37.89 % (2839)	31.50 % (1197)		32.85 % (2234)	32.62 % (1109)	33.09 % (1125)	
Stage 2	55.98 % (6322)	54.16 % (4058)	59.58 % (2264)		58.71 % (3992)	59.15 % (2011)	58.26 % (1981)	
Stage 3	8.28 % (935)	7.95 % (596)	8.92 % (339)		8.44 % (574)	8.24 % (280)	8.65 % (294)	
Disease characteristics (%, n)								
Duration of DM				<0.001*				0.652
<5 years	63.91 % (7217)	59.72 % (4475)	72.16 % (2742)		70.03 % (4762)	70.06 % (2382)	70.00 % (2380)	
5–10 years	23.84 % (2692)	26.37 % (1976)	18.84 % (716)		20.50 % (1394)	20.76 % (706)	20.24 % (688)	
>10 years	12.26 % (1384)	13.91 % (1042)	9.00 % (342)		9.47 % (644)	9.18 % (312)	9.76 % (332)	
Hypertension				<0.001*				0.669
No	28.30 % (3196)	31.59 % (2367)	21.82 % (829)		23.81 % (1619)	24.03 % (817)	23.59 % (802)	
Yes	71.70 % (8097)	68.41 % (5126)	78.18 % (2971)		76.19 % (5181)	75.97 % (2583)	76.41 % (2598)	
Treatment modalities (%, n)								
Use of anti-hypertensive drugs				<0.001*				0.956
No	29.33 % (3312)	31.06 % (2327)	25.92 % (985)		26.41 % (1796)	26.38 % (897)	26.44 % (899)	
Yes	70.67 % (7981)	68.94 % (5166)	74.08 % (2815)		73.59 % (5004)	73.62 % (2503)	73.56 % (2501)	
Use of lipid-lowering agents				0.032*				0.699
No	95.71 % (10809)	95.42 % (7150)	96.29 % (3659)		96.32 % (6550)	96.41 % (3278)	96.24 % (3272)	
Yes	4.29 % (484)	4.58 % (343)	3.71 % (141)		3.68 % (250)	3.59 % (122)	3.76 % (128)	

*MM* metformin monotherapy, *HbA1c* haemoglobin A1c, *SBP* systolic blood pressure, *DBP* diastolic blood pressure, *LDL-C* low density lipoprotein—cholesterol, *TC* Total cholesterol, *HDL-C* high density lipoprotein-cholesterol, BMI body mass index, eGFR estimated glomerular filtration rate, *DM* diabetes mellitus, *CKD* chronic kidney disease

Stage 1 CKD (eGFR ≥90 ml/min/1.73 m^2^); Stage 2 CKD (eGFR ≥60 & <90 ml/min/1.73 m^2^); Stage 3 CKD (eGFR ≥30 & <60 ml/min/1.73 m^2^)

* Significant with p value < 0.05 by Chi-square test or t test as appropriate

Table 2 compares the clinical parameters between MM and control groups in the full and propensity score-matched cohorts. MM group showed significant improvement in HbA1c, SBP, DBP, LDL-C, TC/HDL-C, and BMI when compared to control group in matched cohort, with the addition of TG lowering in full cohort. Table 3 compares the change in CKD staging, with the “post” reading taken as on or before the date of last follow-up, between MM and control groups, and patients in MM group were more likely to progress from lower CKD stage to higher CKD stage compared with control group.

**Table 2:** Clinical parameter comparisons between baseline and post

Clinical parameters (Mean ± SD)	Full cohort with complete case				Propensity score-matched cohort			
	MM group paired diff.^a^ (N = 7493)	Control group paired diff.^a^ (N = 3800)	Diff. in diff. (MM group—control group)	P value	MM group paired diff.^a^ (N = 3400)	Control group paired diff.^a^ (N = 3400)	Diff. in diff. (MM group—control group)	P value
HbA1c, %	−0.04 ± 1.08	0.29 ± 0.68	−0.32	<0.001*	0.19 ± 0.79	0.28 ± 0.69	−0.09	<0.001*
SBP, mmHg	−2.86 ± 19.33	−1.91 ± 19.04	−0.95	0.013*	−3.07 ± 19.40	−1.95 ± 18.95	−1.12	0.016*
DBP, mmHg	−2.20 ± 10.64	−1.42 ± 10.16	−0.78	<0.001*	−2.25 ± 10.54	−1.30 ± 10.15	−0.95	<0.001*
LDL-C, mmol/L	−0.58 ± 0.90	−0.48 ± 0.89	−0.10	<0.001*	−0.71 ± 0.92	−0.44 ± 0.87	−0.27	<0.001*
TC/HDL-C ratio	−0.69 ± 1.04	−0.60 ± 1.22	−0.09	<0.001*	−0.78 ± 1.07	−0.56 ± 1.01	−0.23	<0.001*
Triglyceride, mmol/L	−0.21 ± 0.84	−0.14 ± 0.75	−0.07	<0.001*	−0.18 ± 0.73	−0.14 ± 0.77	−0.03	0.059
BMI, kg/m^2^	−0.30 ± 1.55	−0.05 ± 1.46	−0.25	<0.001*	−0.19 ± 1.54	−0.08 ± 1.43	−0.11	0.002*

*MM* metformin monotherapy, *HbA1c* haemoglobin A1c, *SBP* systolic blood pressure, *DBP* diastolic blood pressure, *LDL-C* low density lipoprotein-cholesterol, *TC* total cholesterol, *HDL-C* high density lipoprotein-cholesterol, *BMI* body mass index, DM diabetes mellitus

* Significant with p value <0.05 by independent t test

^a^Paired difference (Post—baseline)

**Table 3:** Comparisons of stage of chronic kidney disease between baseline and post

Stage of CKD (%, n/N)	Full cohort with complete case			Propensity score-matched cohort		
	MM group	Control group	P value	MM group	Control group	P value
Stage 1 (baseline) to stage 2 or above (post)	21.98 % (624/2839)	21.72 % (260/1197)	0.856	23.26 % (258/1109)	21.07 % (237/1125)	0.211
Stage 2 (baseline) to stage 3 or above (post)	10.62 % (431/4058)	6.89 % (156/2 264)	<0.001*	11.09 % (223/2011)	6.66 % (132/1981)	<0.001*
Stage 3 (baseline) to stage 4 or above (post)	10.74 % (64/596)	9.73 % (33/339)	0.629	11.43 % (32/280)	9.18 % (27/294)	0.376
Total	14.93 % (1119/7493)	11.82 % (449/3800)	<0.001*	15.09 % (513/3400)	11.65 % (396/3400)	<0.001*

*MM* metformin monotherapy, *CKD* chronic kidney disease, *eGFR* estimated glomerular filtration rate

Stage 1 CKD (eGFR ≥90 ml/min/1.73 m^2^); Stage 2 CKD (eGFR ≥60 & <90 ml/min/1.73 m^2^); Stage 3 CKD (eGFR ≥30 & <60 ml/min/1.73 m^2^)

Stage 4 CKD (eGFR ≥15 & <30 ml/min/1.73 m^2^); Stage 5 CKD (eGFR <15 ml/min/1.73 m^2^)

* Significant with p value <0.05 by Chi-square test

**Fig. 2:**
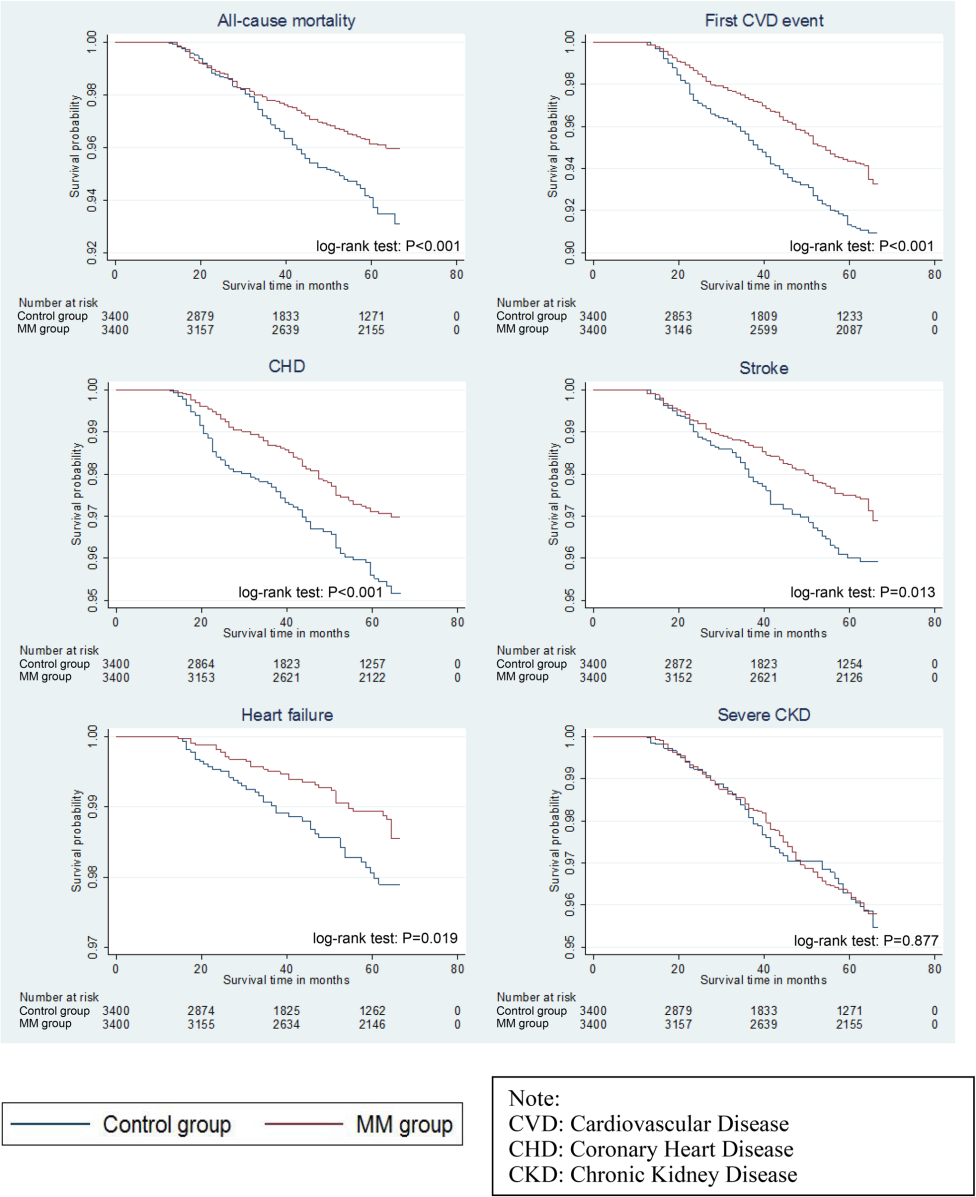
Kaplan–Meier survival curves of outcomes. *MM* metformin monotherapy, *CVD* cardiovascular disease, *CHD* coronary heart disease, *CKD* chronic kidney disease

Table 4 and Fig. 2 show the number and incidence rates of all-cause mortality and CVD events for the two cohorts together with the Kaplan–Meier survival curves. MM group had lower incidence rates in all-cause mortality and CVD events than control group and were obtained in both full cohort and propensity score-matched cohort. Under the propensity score-matched cohort, the median follow-up period for MM group was longer (62.5 months) than the control group (43.5–44.5 months). MM group had an incidence rate of 7.5 deaths per 1000 person-years in a median follow-up period of 62.5 months whereas control group had 11.1 deaths per 1000 person-years in a median follow-up period of 44.5 months. Similarly, MM group had a CVD incidence rate of 11.3 per 1000 person-years in a median follow-up period of 62.5 months whereas control group had a CVD incidence rate of 16.2 per 1000 person-years in a median follow-up period of 43.5 months. Further breakdown of the CVD events into CHD, stroke and heart failure also showed results in line with that of the all-cause mortality and CVD event. However, more severe CKD incidents were observed in the MM group (7.4 per 1000 person-years) than the control group (6.9 per 1000 person-years).

**Table 4:** Number and incidence rates of all-cause mortality, CVD event and severe chronic kidney disease

Event	Cumulative incidence		Incidence rate (cases/1000 person-years)		Person-years	Median follow-up periods (months)
	No. of event	Rate (%)	Estimate	95 % CI^a^		
Full cohort (N = 11,293)						
All-cause mortality	397	3.52	8.713	(7.877–9.614)	45,561.5	60.5
CVD	610	5.40	13.527	(12.474–14.644)	45,095.8	58.5
CHD	294	2.60	6.482	(5.763–7.267)	45,353.5	59.5
Stroke	266	2.36	5.865	(5.182–6.614)	45,351.9	59.5
Heart failure	139	1.23	3.055	(2.569–3.608)	45,493.0	60.5
Severe CKD	333	2.95	5.266	(4.716–5.864)	63,230.1	60.5
MM group (N = 7493)						
All-cause Mortality	240	3.20	7.591	(6.661–8.615)	31,615.0	62.5
CVD	382	5.10	12.204	(11.011–13.492)	31,300.8	61.5
CHD	176	2.35	5.591	(4.796–6.481)	31,477.2	61.5
Stroke	170	2.27	5.403	(4.621–6.279)	31,466.3	61.5
Heart failure	85	1.13	2.692	(2.150–3.328)	31,580.7	62.5
Severe CKD	230	3.07	7.275	(6.365–8.278)	31,615.0	62.5
Control group (N = 3800)						
All-cause Mortality	157	4.13	11.257	(9.565–13.162)	13,946.5	45.5
CVD	228	6.00	16.528	(14.452–18.818)	13,795.0	44.5
CHD	118	3.11	8.504	(7.039–10.184)	13,876.3	45
Stroke	96	2.53	6.914	(5.600–8.443)	13,885.6	44.5
Heart failure	54	1.42	3.881	(2.916–5.064)	13,912.3	45.5
Severe CKD	103	2.71	7.385	(6.028–8.957)	13,946.5	45.5
Propensity score-matched cohort (N = 6800)						
All-cause mortality	249	3.66	9.104	(8.009–10.308)	27,349.6	60.5
CVD	366	5.38	13.527	(12.177–14.987)	27,056.3	58.5
CHD	183	2.69	6.724	(5.785–7.772)	27,217.4	59.5
Stroke	162	2.38	5.951	(5.070–6.942)	27,221.0	59.5
Heart failure	77	1.13	2.821	(2.226–3.525)	27,298.1	60.5
Severe CKD	197	2.90	7.203	(6.232–8.282)	27,349.6	60.5
MM group (N = 3400)						
All-cause mortality	113	3.32	7.493	(6.175–9.008)	15,081.4	62.5
CVD	169	4.97	11.330	(9.686–13.173)	14,915.8	62.5
CHD	81	2.38	5.398	(4.287–6.710)	15,004.7	62.5
Stroke	76	2.24	5.065	(3.990–6.339)	15,006.4	62.5
Heart failure	34	1.00	2.257	(1.563–3.154)	15,062.0	62.5
Severe CKD	112	3.29	7.426	(6.115–8.936)	15,081.4	62.5
Control group (N = 3400)						
All-cause mortality	136	4.00	11.086	(9.301–13.113)	12,268.2	44.5
CVD	197	5.79	16.227	(14.040–18.658)	12,140.6	43.5
CHD	102	3.00	8.352	(6.810–10.139)	12,212.8	43.5
Stroke	86	2.53	7.041	(5.632–8.695)	12,214.6	43.5
Heart failure	43	1.26	3.514	(2.543–4.734)	12,236.1	43.5
Severe CKD	85	2.50	6.929	(5.534–8.567)	12,268.2	44.5

*MM* metformin monotherapy, *CVD* cardiovascular disease, *CHD* coronary heart disease, *eGFR* estimated glomerular filtration rate, *DM* diabetes mellitus, *CI* confidence interval, *CKD* chronic kidney disease

^a^The 95 % CI was constructed based on Poisson distribution

Multivariable Cox proportional hazard regressions were performed on the dependent variables of all-cause mortality, CVD event and severe CKD, and results for both full and propensity score-matched cohorts are shown in Table 5. The range of variance inflation factors was from 1 to 3.21 which indicated absence of multi-collinearity and the proportional hazard. Random scattered points were observed from the scaled Schoenfeld residual plots which satisfied the proportional hazard assumption of Cox models. The full cohort and propensity score-matched cohort demonstrated similar results. After propensity score matching, there was a 29.5 % risk reduction of all-cause mortality in MM group compared to control group and the difference in their survival time was highly significant (P < 0.001) by the log-rank test. Moreover, patients in MM group had around 30–35 % risk reduction in the incidence of all the CVD events, including CHD and stroke except heart failure, when compared to the control group. Log-rank test also suggested that there were significant differences (P < 0.001 for CVD event and CHD; P = 0.013 for stroke) in the survival times of all the CVD events between the two groups. For heart failure, log-rank test showed significant difference between the two groups (P = 0.019) but the risk of incident heart failure in Cox model showed no significant difference between MM and control groups. Moreover, the risk of severe CKD was increased by 16.3 % (though statistically insignificant) in MM group when compared to control group. This was also in line with the results in log-rank test (P = 0.877).

**Table 5:** Multivariable Cox proportional hazard regression all-cause mortality, CVD event and severe chronic kidney disease

	MM group comparing with control group			Harrell’s C-statistic
	HR^a^	95 % CI	P value	
Full cohort (N = 11,293)				
All-cause mortality	0.725	(0.584–0.901)	0.004*	0.806 (0.783–0.828)
CVD	0.726	(0.609–0.866)	<0.001*	0.731 (0.711–0.752)
CHD	0.670	(0.521–0.862)	0.002*	0.727 (0.698–0.756)
Stroke	0.750	(0.573–0.982)	0.036*	0.734 (0.704–0.765)
Heart failure	0.795	(0.551–1.147)	0.221	0.872 (0.844–0.899)
Chronic kidney disease (eGFR <30 ml/min/1.73 m^2^)	1.076	(0.838–1.381)	0.565	0.848 (0.827–0.870)
Propensity score-matched cohort (N = 6800)				
All-cause mortality	0.705	(0.547–0.908)	0.007*	0.809 (0.783–0.836)
CVD	0.684	(0.556–0.842)	<0.001*	0.731 (0.705–0.756)
CHD	0.645	(0.480–0.866)	0.004*	0.720 (0.683–0.758)
Stroke	0.698	(0.511–0.954)	0.024*	0.754 (0.719–0.790)
Heart failure	0.688	(0.435–1.086)	0.109	0.865 (0.825–0.906)
Chronic kidney disease (eGFR <30 ml/min/1.73 m^2^)	1.163	(0.874–1.549)	0.300	0.854 (0.826–0.881)

*MM* metformin monotherapy, *CVD* cardiovascular disease, *CHD* coronary heart disease, *eGFR* estimated glomerular filtration rate, *DM* diabetes mellitus, *HR* hazard ratio, *CI* confidence interval

* p value <0.05

^a^HR >1 indicates greater risk for death

The details of the sensitivity analysis are shown in Additional file 1: Table S1 and the corresponding Kaplan–Meier survival curves are plotted in Additional file 1: Fig. S1a, b. If the exclusion of outcome events occurring within 1 year after baseline was considered, the results were better than the main analysis with 33.4, 27.6, 25.7, 30.0 and 36.0 % significant risk reduction of all-cause mortality, CVD event, CHD, stroke and even heart failure in MM group compared to the control group. However, the effect of Metformin was reduced if the intention-to-treat approach was adopted. Although MM group had a risk reduction of 10–20 % in all of the outcome events except chronic kidney disease compared to the control group, only the risk reductions of CVD event and CHD reached statistically significance.

## Discussions

This article is by far the latest and largest head-to-head comparison, between MM and diabetic patients not receiving any medications for their DM, on the clinical outcomes of Chinese diabetic patients at the primary care level. This study showed that diabetic patients who were started with MM showed improvement in many of the clinical parameters including HbA1c, SBP, DBP, LDL-C, TC/HDL-C, and BMI, and a reduction in all-cause mortality and CVD events when compared to lifestyle changes alone. Metformin is recommended as the first-line OAD drug worldwide unless contraindicated. The most difficult decision for the primary care doctors is to decide if and when the diabetic patient needs to start metformin at the early stage of management of their diabetes in order to modify the disease course and enhance the prognosis. Findings from this study can give more light to primary care doctors on making such decision. It was worthwhile to note that at the baseline, about half of our eligible diabetic patients (24,350 out of 49,478) were not prescribed any anti-diabetic medications, and the number was comparable to that on metformin monotherapy (25,128 out of 49,478). The underlying reasons are worth exploring. Some patients may want to try lifestyle modifications first and to see if there are any improvements in their control of DM before using medications. Patients’ concern of the side-effects of the drugs, drug compliance issues, and lifelong drug therapy are barriers to patients starting metformin. Some patients may seek help from alternative medicine or Chinese herbs, which is not uncommon in Chinese community as traditional Chinese medicine has its own school of theory on DM [26–28]. The financial influence on the decision to prescribe metformin in our study population was minimal as patients under primary care in the public system only needed to pay HKD 45 [USD 5.78] for the consultation fee and there was no additional drug fee for the metformin. Doctors, on the other hand, may use “non-pharmacological” approach of management as an incentive to make their patients more adhere to healthier lifestyle modifications through diet control and regular exercise. Patient may depend on metformin rather than diet and exercise if they knew that the medications was helping them [29].

Although lifestyle modifications are supposed to be harm-free, it is generally a vague concept, and patients may not be able to comply closely with these lifestyle modifications [30]. Patients who are prescribed metformin were usually advised to continue with the lifestyle modifications that were relevant to DM. Patients on metformin had reduced incidence rate of all-cause mortality, CVD events (including CHD, stroke, and heart failure), and severe CKD. The longer median follow-up periods (43.5–44.5 months) for control group than the MM group (62.5 months) suggested that control group had a higher chance to have a change in their condition (either the control of DM or an event) that warrants change of their management of DM. When the severity of DM, as reflected by HbA1c, and other factors were controlled, a patient on metformin had 29.8 % lower risk of having all-cause mortality, and 29.6 % lower risk of having any of the CVD event when compared to a patient on lifestyle modifications alone. These risk reductions may be partially attributed by the coherent improvement in SBP, DBP, LDL-C, TC/HDL-C, TG and BMI. HbA1c, the indicator used to reflect the control of DM in the past 2–3 months, on the contrary showed an elevation. Nevertheless, the rise in HbA1c was significantly less than that of control group. The matched cohort showed that MM group had a net less of 0.09 % in HbA1c compared to control group during the follow-up period. With reference to the United Kingdom Prospective Diabetes Study (UKPDS), every 1 % reduction in HbA1c was associated with reduction in risk of stroke by 12 %, CHD by 9.9 %, heart failure by 16 % and all-cause mortality by 14 % [31, 32], our study showed that patients in MM group may well have a reduction in stroke, CHD, heart failure, and all-cause mortality by 30.2, 35.5, 31.2 and 29.5 %, respectively, when compared to control group. Metformin was suggested to be able to modify LDL-C and TG, and may be associated with weight loss [33–35]. In this study, patients in MM group also achieved a significant weight loss (a significant 0.15 kg/m^2^ less) when compared to control group, and a significant of 0.29 mmol/L drop in LDL-C and 0.02 mmol/L TG. This may explain why patients in MM group had a reduced risk for CVD despite the absence of HbA1c reduction. The other reason may be the direct protective effects of the heart by metformin [36–42]. Metformin was found capable to activate AMP-activated protein kinase thus protecting human coronary artery endothelial cells against diabetic lipoapoptosis in experimental setting [37]. Metformin was found to improve glycocalyx barrier properties in db/db mice [38], attenuate Ang-II-induced atheromatous plaque formation and aortic aneurysm in ApoE(−/−) mice [39]. Treatment with metformin significantly attenuates neointimal hyperplasia in fructose-induced insulin resistant rats [40] and myocardial remodeling and neutrophil recruitment after myocardial infarction in rats [41]. In addition, metformin monotherapy was associated with a decreased risk of atrial fibrillations in patients with type 2 DM, probably via attenuation of atrial cell tachycardia-induced myolysis and oxidative stress [42]. The beneficial effects of metformin obtained from these studies reinforced our study findings on the cardiovascular protective effects of metformin use on patients with type 2 DM. Some studies also advocated the early use of metformin in patients with pre-diabetes, as it could potentially postpone the development of DM and reduce the associated cardiovascular risk [43–45]. Further studies are needed to explore the relationship between metformin and CVDs.

Although patients on metformin monotherapy was at a higher risk of having eGFR <30 ml/min/1.73 m^2^, Patients in MM group were found to be non-inferior in risk of severe CKD compared to those in control group. However, the breakdown analysis showed that a patient on metformin was more prone to progress towards poor renal function in the CKD stages (MM group 15.5 % vs control group 11.5 %). Caution should be implemented when prescribing metformin and renal function should be regularly monitored.

In view of the overall significant benefit of metformin use early in the course of diabetic patient management, primary care doctors should discuss with their patients in details about the early initiation of metformin for their long-term management plan of DM. At the same time, the role of lifestyle modifications, including DM diet, regular exercise, and weight control, are equally important as patients with chronic disease are now encouraged to be more self-empowered or self-enabled when living with their chronic diseases [14]. Doctors should also monitor their renal function regularly, and be alert if a patient is having any metformin-related side-effects, like vitamin B12 deficiency presentations [46].

### Strengths and limitations of this study

The large number of diabetic patients involved in this study and the long period of follow-up helped reflect the effect of metformin monotherapy and lifestyle modifications on the outcomes of diabetic patients. The propensity score-matched cohort controlled many of the confounding factors like age of patient, severity of DM at baseline, etc. The consistency of most findings from both full cohort with complete case and propensity score-matched cohort analytical approaches strongly suggested that use of metformin is comparatively beneficial to diabetic patients in terms of their clinical parameters and clinical outcomes.

Limitations of the study included, firstly, the incomplete data of the baseline covariates. Significant proportions of patients in MM and control groups were dropped out from the analysis because they lacked the baseline covariates. Although the propensity score matching can help to balance the baseline characteristics between the MM and control groups, this greatly reduced the sample size, especially the MM group (from 7493 to 3400), after matching. Secondly, we lacked some fine details on the use of metformin, including the change in exact dosage of metformin of each individual patient taking it. Drug compliance to metformin could not be assessed in our study. The side-effects of metformin and the contraindication to metformin had also not been taken into account, but an attempt was tried to reveal if metformin was related to worsening of renal function by monitoring the eGFR and the CKD staging. Further studies can focus on metformin monotherapy versus other class of anti-diabetic drugs, or the effectiveness of metformin combination therapy in the management of diabetic patients. Lastly, our study relied on the ICPC-2 and ICD-9-CM diagnosis coding in identifying all the outcome events. However, there were no prior studies conducted to evaluate the accuracy and completeness of these coding methods. Possible misclassification bias may have occurred.

## Conclusions

Type 2 diabetic patients who were started on metformin monotherapy showed improvement in many of the clinical parameters and a reduction in all-cause mortality and CVD events when compared with lifestyle modifications alone. If there is no contraindication and if tolerated, diabetic patients should be prescribed with metformin early in the course of the management plan to minimize their risk of having the cardiovascular events and mortality in the long run. However, renal function should be regularly monitored.

## Additional file

10.1186/s12933-015-0304-2 Supplementary information

## Abbreviations

BMI: body mass index
CI: confidence interval
CHD: coronary heart disease
CKD: chronic kidney disease
CVD: cardiovascular disease
DBP: diastolic blood pressure
DM: diabetes mellitus
eGFR: estimated glomerular filtration rate
ESRF: end stage renal failure
HA: Hospital Authority
HbA1c: haemoglobin A1c
HDL-C: high-density lipoprotein-cholesterol
HR: hazard ratio
ICD-9-CM: International Classification of Diseases, Ninth Edition, Clinical Modification
ICPC-2: International Classification of Primary Care-2
LDL-C: low-density lipoprotein-cholesterol
MM: metformin monotherapy
OAD: oral anti-diabetic
STDR: sight-threatening diabetic retinopathy
SBP: systolic blood pressure
TC: total cholesterol
TG: triglyceride
T2DM: type 2 diabetes mellitus
UKPDS: United Kingdom Prospective Diabetes Study
