# Supplementary material for: Effect of metformin monotherapy on cardiovascular diseases and mortality: a retrospective cohort study on Chinese type 2 diabetes mellitus patients
Source: Cardiovasc Diabetol. 2015 Oct 9;14:137. doi: 10.1186/s12933-015-0304-2 (PMC4600251; doi:10.1186/s12933-015-0304-2)

Table S1. Sensitivity analysis for scenario: (1) patients with the exclusion of outcome events occurring within 1 year after study beginning; and (2) using intention-to-treat approach

| Propensity Score-Matched Cohort | MM group comparing with control group | | | Harrell's C-statistic |
| --- | --- | --- | --- | --- |
|  | HR^†^ | 95%CI | P-value |  |
| **Scenario 1 (N = 6 898)** |  |  |  |  |
| All-cause Mortality | 0.666 | (0.520,0.854) | 0.001* | 0.808 (0.781,0.836) |
| CVD | 0.724 | (0.594,0.881) | 0.001* | 0.727 (0.701,0.752) |
| CHD | 0.743 | (0.564,0.977) | 0.033* | 0.728 (0.692,0.764) |
| Stroke | 0.700 | (0.520,0.943) | 0.019* | 0.730 (0.694,0.766) |
| Heart Failure | 0.640 | (0.415,0.988) | 0.044* | 0.890 (0.863,0.917) |
| Chronic kidney disease (eGFR < 30ml/min/1.73m^2^) | 1.050 | (0.799,1.380) | 0.725 | 0.874 (0.851,0.897) |
|  |  |  |  |  |
| **Scenario 2 (N = 18 050)** |  |  |  |  |
| All-cause Mortality | 0.905 | (0.773,1.059) | 0.212 | 0.784 (0.766,0.802) |
| CVD | 0.846 | (0.745,0.960) | 0.010* | 0.712 (0.695,0.728) |
| CHD | 0.800 | (0.664,0.963) | 0.018* | 0.696 (0.672,0.720) |
| Stroke | 0.930 | (0.766,1.130) | 0.465 | 0.709 (0.684,0.734) |
| Heart Failure | 0.910 | (0.693,1.194) | 0.496 | 0.837 (0.808,0.866) |
| Chronic kidney disease (eGFR < 30ml/min/1.73m^2^) | 1.029 | (0.870,1.218) | 0.736 | 0.839 (0.821,0.856) |

Notes:

MM = Metformin Monotherapy; CVD = Cardiovascular Disease; CHD = Coronary Heart Disease; eGFR = Estimated Glomerular Filtration Rate; DM = Diabetes Mellitus; HR = Hazard Ratio; CI = Confidence Interval

* p-value < 0.05

^†^ HR > 1 indicates greater risk for death

Figure S1a: Kaplan-Meier survival curves of outcomes for patients with the exclusion of outcome events occurring within 1 year after study beginning


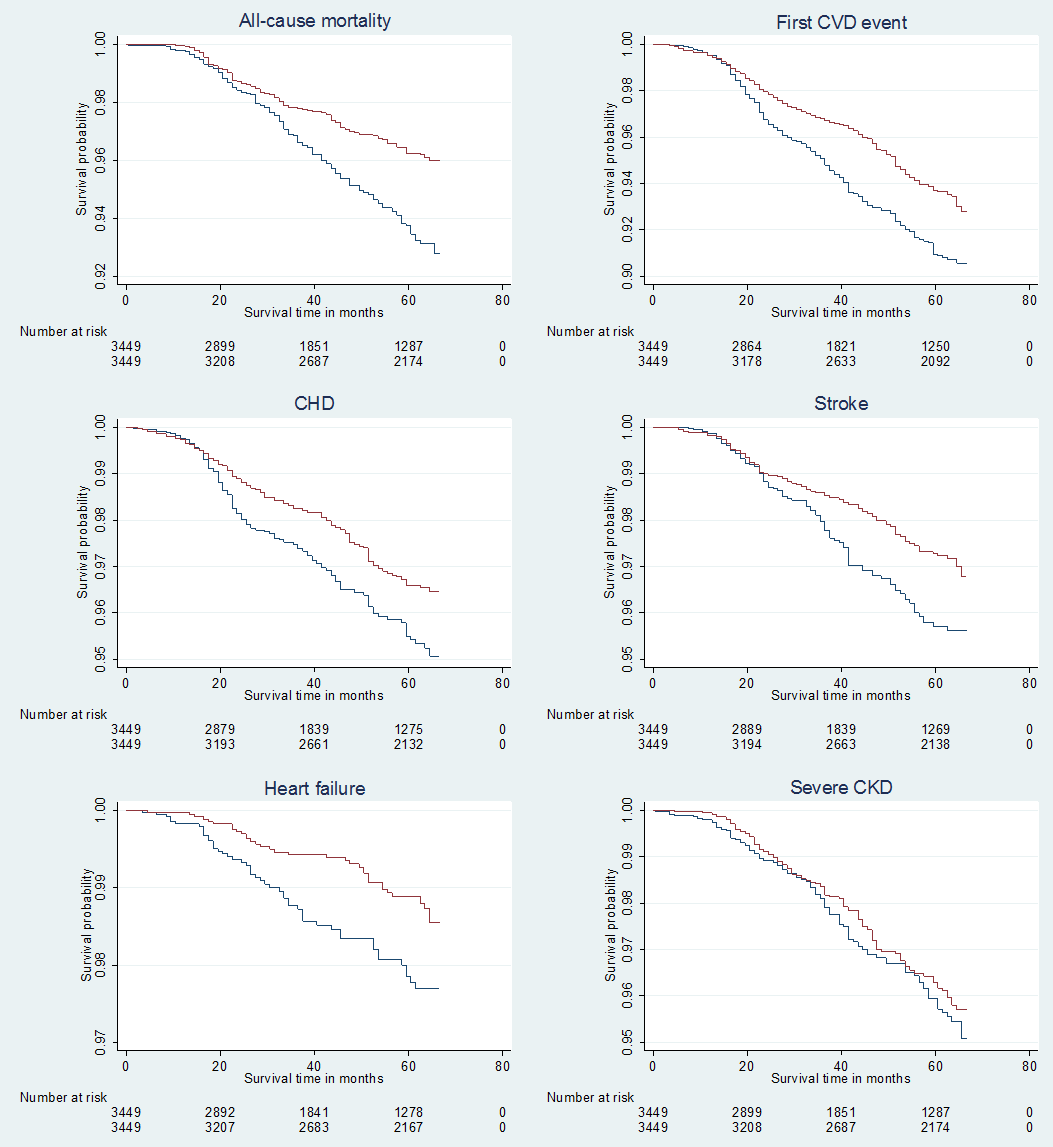


Control group

MM group

Control group

MM group

Control group

MM group

Control group

MM group

Control group

MM group

Control group

MM group

log-rank test: P=0.425

log-rank test: P=0.004

log-rank test: P=0.009

log-rank test: P=0.013

log-rank test: P<0.001

log-rank test: P<0.001

Note:

CVD: Cardiovascular Disease

CHD: Coronary Heart Disease

CKD: Chronic Kidney Disease


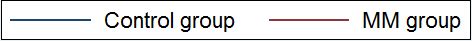


Figure S1b: Kaplan-Meier survival curves of outcomes using intention-to-treat approach


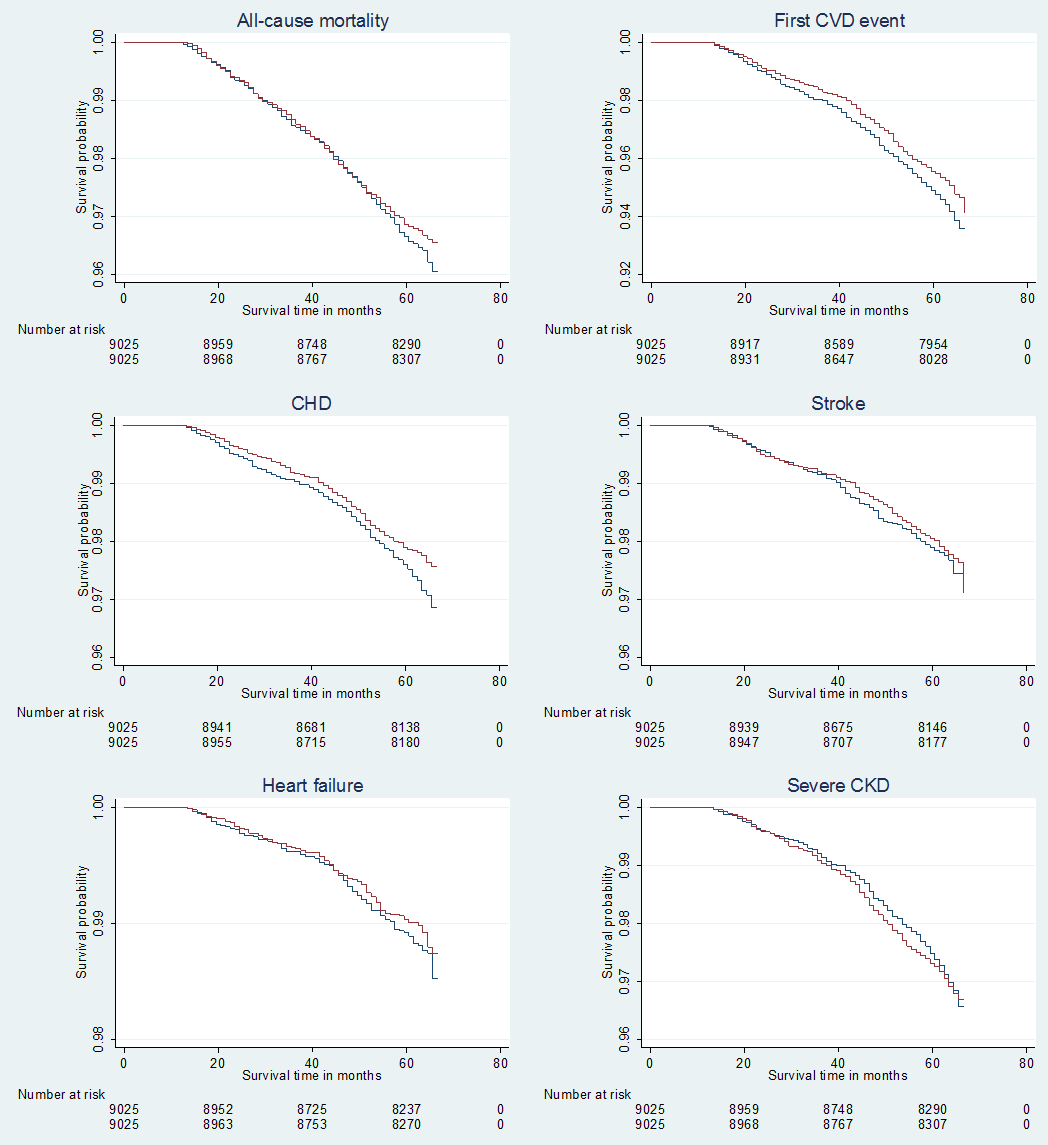


Control group

MM group

Control group

MM group

Control group

MM group

Control group

MM group

Control group

MM group

Control group

MM group

log-rank test: P=0.891

log-rank test: P=0.408

log-rank test: P=0.018

log-rank test: P=0.486

log-rank test: P=0.009

log-rank test: P=0.215

Note:

CVD: Cardiovascular Disease

CHD: Coronary Heart Disease

CKD: Chronic Kidney Disease


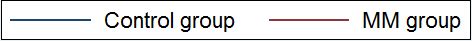

Supplement: Supplementary file 1 — 10.1186/s12933-015-0304-2 Supplementary information [file 12933_2015_304_MOESM1_ESM.docx]
